# Moderate Exercise Mitigates the Detrimental Effects of Aging on Tendon Stem Cells

**DOI:** 10.1371/journal.pone.0130454

**Published:** 2015-06-18

**Authors:** Jianying Zhang, James H-C. Wang

**Affiliations:** 1 MechanoBiology Laboratory, Department of Orthopaedic Surgery, University of Pittsburgh, Pittsburgh, Pennsylvania, United States of America; 2 Department of Bioengineering, University of Pittsburgh, Pittsburgh, Pennsylvania, United States of America; 3 Department of Mechanical Engineering and Materials Science, University of Pittsburgh, Pittsburgh, Pennsylvania, United States of America; 4 Department of Physical Medicine and Rehabilitation, University of Pittsburgh, Pittsburgh, Pennsylvania, United States of America; Queen Mary University of London, UNITED KINGDOM

## Abstract

Aging is known to cause tendon degeneration whereas moderate exercise imparts beneficial effects on tendons. Since stem cells play a vital role in maintaining tissue integrity, in this study we aimed to define the effects of aging and moderate exercise on tendon stem/progenitor cells (TSCs) using *in vitro* and *in vivo* models. TSCs derived from aging mice (9 and 24 months) proliferated significantly slower than TSCs obtained from young mice (2.5 and 5 months). In addition, expression of the stem cell markers Oct-4, nucleostemin (NS), Sca-1 and SSEA-1 in TSCs decreased in an age-dependent manner. Interestingly, moderate mechanical stretching (4%) of aging TSCs *in vitro* significantly increased the expression of the stem cell marker, NS, but 8% stretching decreased NS expression. Similarly, 4% mechanical stretching increased the expression of Nanog, another stem cell marker, and the tenocyte-related genes, collagen I and tenomodulin. However, 8% stretching increased expression of the non-tenocyte-related genes, LPL, Sox-9 and Runx-2, while 4% stretching had minimal effects on the expression of these genes. In the *in vivo* study, moderate treadmill running (MTR) of aging mice (9 months) resulted in the increased proliferation rate of aging TSCs in culture, decreased lipid deposition, proteoglycan accumulation and calcification, and increased the expression of NS in the patellar tendons. These findings indicate that while aging impairs the proliferative ability of TSCs and reduces their stemness, moderate exercise can mitigate the deleterious effects of aging on TSCs and therefore may be responsible for decreased aging-induced tendon degeneration.

## Introduction

Tendons are fibrous connective tissue, largely made of collagens, proteoglycans, glycoproteins, water and cells. Their primary function is to transmit muscular forces to bones, thereby enabling joint movement. Therefore, tendons constantly experience mechanical loading in varying degrees. Traditionally, tendons were considered to contain only one cell type, the tenocytes, which are resident fibroblast-like cells that maintain tendon integrity, remodeling and repair [1]. However, a new tendon cell type, termed tendon stem/progenitor cells (TSCs), has been identified in recent years in humans, rabbits, mice, and rats [2–4]. TSCs differ from tenocytes in their ability to proliferate and self-renew, as well as in their multi-differentiation potential, which allows them to differentiate into different cell types such as adipocytes, chondrocytes, and osteocytes, in addition to differentiation into tenocytes [4].

In recent years, a few studies have been performed to better understand the cellular and molecular mechanisms responsible for the effects of aging on tendons [5–7]. In general, aging slowly lowers the functional competence of the human body, largely due to the damages in DNA, changes in the cellular microenvironments of the body and epigenetic regulation [8]. In tendons, aging increases the nucleus to cytoplasm ratio and lipid deposition, but decreases vascularization and tendon matrix integrity, and alters tendon cell’s response to cellular stimuli [9]. In addition, aging also reduces the number of tendon cells and decreases their activity [10–12] thereby depleting the resources required to repair injured tendons. Consequently, there is a steady decline in the ability of tendons to repair its injuries over time [13]. Through these changes aging reduces the mechanical strength of tendons and makes them susceptible to injuries, thus lowering the quality of life of the aging population and increasing the healthcare cost.

While aging generally causes detrimental effects on tendons, exercise is known to exert beneficial effects on tendons. For example, exercise improves the mechanical strength of the rabbit peroneus brevis tendon [14,15]. In mice that ran on a treadmill for one week, the number and size of collagen fibrils as well as the cross-sectional area of the digital flexor tendons increased when compared to mice at rest [16,17]. Similarly, in human Achilles tendons, physical training promoted the concentration of collagen propeptide (PICP) and decreased collagen degradation product (ICTP) indicating a net increase in collagen I due to exercise [18]. Indeed, long-term exercise is known to increase tendon-tissue mass, collagen content, cross-sectional area, load to failure, ultimate tensile strength, and weight-to-length ratio [19,20]. Thus, exercise positively influences the structure and mechanical strength of tendons. However, the role of TSCs in aging- and exercise-induced changes in tendons is not well understood.

Therefore, to explore the TSC-based mechanisms responsible for the beneficial effects of exercise on aging tendons, we tested two hypotheses in this study: i) aging impairs TSC function in tendons, and ii) moderate exercise revives impaired TSC function and thereby exerts beneficial effects on aging tendons. We tested the first hypothesis on mouse TSCs *in vitro* and determined the effects of aging on TSC proliferation and stem cell marker expression. In addition, we investigated the presence of non-tendinous tissues in young and aging mice *in vivo* and the multi-differentiation potential of TSCs from young and aging mice *in vitro*. To test the second hypothesis we used *in vitro* and *in vivo* "exercise" models and determined the effects of moderate exercise on aging TSCs, in terms of their morphology, proliferation, and tenocyte and non-tenocyte-related gene expression, as well as on tendon degeneration due to aging.

## Materials and Methods

### Isolation of TSCs from young and aging mice

All animal protocols used in this study were approved by the University of Pittsburgh IACUC. To determine the effects of aging on TSCs, we first isolated them from the patellar tendons from young (2.5, and 5 months) and aging (9, and 24 months) mice and cultured them using a protocol described previously [4]. After 2 weeks in culture at 37°C and 5% CO_2_, colony development was monitored and cell morphology was observed through a microscope. When cells reached ~90% confluence they were detached from the plates by trypsinization with 0.05% trypsin and counted on an automated cell counter (Cellometer, Nexcelom, Lawrence, MA). On average, aging TSCs from 9 months old mice remained in culture for 3.5 times longer than the 2.5 months old TSCs to reach ~90% confluence.

### Determination of TSC proliferation *in vitro*

Proliferation of mouse TSCs isolated from the various age groups was calculated based on the population doubling time (PDT), which is the total time required by cells to double in number. PDT was calculated from the formula log_2_[Nc/N_0_], where N_0_ refers to the total cell number during seeding, and Nc is the total cell number at confluence [4].

### Stem cell marker expression in TSCs from young and aging mice

Immunocytochemical staining was used to determine the stemness of TSCs isolated from mice in the various age groups. The stem cell markers, Oct-4, NS, Sca-1, and SSEA-1 were analyzed as described previously [21].

### Semi-quantitation of stem cell marker staining in TSCs from young and aging mice

To quantify stem cell marker staining *in vitro*, we used a semi-quantitation method. First, we obtained seven randomly selected images of TSCs stained for each stem cell marker using a camera attached to a fluorescence microscope. Then, positive staining in each image was identified using SPOT (Diagnostic Instruments, Inc., Sterling Heights, MI). The percentage of positive staining in each was estimated by dividing the number of positively stained cells by the total number of cells in the microscopic field, and multiplying by 100. The final percentage of positive staining was derived by averaging the values from all seven images.

### Detecting non-tendinous tissues in tendons from young and aging mice

For histochemical analysis, the patellar tendons from young (2.5 months) and aging mice (9 months) were immersed in frozen section medium (Neg 50; Richard-Allan Scientific; Kalamazoo, MI) in pre-labeled base molds and flash frozen in 2-methylbutane chilled in liquid nitrogen. The frozen tissue blocks were sectioned (10 μm), dried overnight at room temperature and stained with Oil Red O, Safranin O or Alizarin Red S as described previously [22].

After staining all samples were rinsed in PBS and examined through a fluorescence microscope (Nikon eclipse, TE2000-U). Image documentation was performed using a CCD (charge-coupled device) camera attached to the microscope. Images were then analyzed using the SPOT imaging software (Diagnostic Instruments, Inc., Sterling Heights, MI).

Semi-quantitation of the positively stained tendon sections was performed by capturing images from 12 different areas in each section at a magnification of 20x. Then, the areas positively stained with Oil Red O, Safranin O or Alizarin Red S were identified using the SPOT imaging software. Finally, the proportion of positive staining was calculated by dividing the sum of positively stained regions by the area in the microscopic field and the average of all 12 values was used to represent the multi-differentiation potential of the respective patellar tendon tissue.

### Cytochemical staining of TSCs from young and aging mice

To assess the differences in multi-differentiation potential of TSCs derived from young (2.5 months) and aging (9 months) mice, cytochemical analyses were performed as previously described [23]. Briefly, TSCs at passage 2 were first grown for 21 days in specific induction media to induce adipogenesis, chondrogenesis and osteogenesis, respectively. Then, TSCs were washed in PBS three times for 5 min each prior to staining with Oil Red O (Millipore, Cat. # 90358), Safranin O solution (Sigma, St. Louis; Cat. # HT904) or Alizarin Red S (Millipore, Cat. # 2003999). After staining, all samples were examined through a microscope (Nikon eclipse, TE2000-U) and the results were documented as images obtained using a CCD camera attached to the microscope. Image analysis was performed using the SPOT imaging software (Diagnostic Instruments, Inc., Sterling Heights, MI).

### Semi-quantitation of cytochemical staining

Cytochemical staining of TSCs was quantified by obtaining 12 images from various locations in the cell culture wells at 20× magnification. Positive staining in each image was analyzed using the SPOT imaging software (Diagnostic Instruments, Inc., Sterling Heights, MI). The percentage of positive staining in each image was estimated as described above (see the section *Semi-quantitation of stem cell marker staining in TSCs from young and aging mice* above).

### Effects of mechanical loading on aging TSCs

To determine the effects of mechanical loading on aging TSCs, we used two previously established experimental models: a) an *in vitro* mechanical loading system where TSCs were stretched to simulate *in vivo* loading conditions [21,24], and b) an *in vivo* mouse treadmill running model [21].

### Application of mechanical loading on TSCs *in vitro*

TSCs were isolated from the patellar tendons of aging mice (9 months old) as described previously [22]. Then, 3×10^5^ cells were seeded into custom-designed, deformable silicone dishes [21] and grown in DMEM + 20% FBS for 24 hrs. Subsequently, TSCs were subjected to cyclic mechanical loading by applying 4% and 8% stretching on the silicone dishes at 0.5 Hz stretching frequency, 2 hrs/day for 3 days. A control group of TSCs was treated under the same conditions except without mechanical loading. At least three replicates were maintained in each of the control, 4%, and 8% stretching groups. Note that these stretching magnitudes represent the so called "clamp-to-clamp" engineering strains, not the strains experienced by cells cultured on the silicone surfaces. The two strain levels are considered as small or moderate and large or excessive mechanical stretching on tendon cells [21,24,25].

### Stem cell marker expression in TSCs after mechanical loading *in vitro*

After mechanical loading, immunocytochemical staining was performed to detect NS expression in aging TSCs from both control and mechanically-loaded groups. The protocol to detect NS expression is essentially as described previously [21]. Goat anti-mouse NS was used as the primary antibody and Cy3-conjugated donkey anti-goat IgG was used as the secondary antibody. Further, semi-quantitation of TSCs that stained positive for NS was also performed as described above (See section *Semi-quantitation of stem cell marker staining in TSCs from young and aging mice* above).

### Gene expression analysis in mechanically stretched TSCs

Gene expression analysis was performed by qRT-PCR on TSCs in the control and mechanically stretched groups (4% and 8%). First, total RNA was extracted from the cells in each group using the RNeasy Mini Kit (Qiagen, Valencia, CA). About 1μg of the total RNA was used as the template to reverse transcribe first-strand cDNA using SuperScript II (Invitrogen, Grand Island, NY) and incubating at 65°C for 5 min, 4°C for 1 min, and 42°C for 50 min. For qRT-PCR, the QIAGEN QuantiTect SYBR Green PCR Kit (QIAGEN, Valencia, CA) was used with 2 μl cDNA (total 100 ng RNA) as the template in a 25 μl PCR reaction volume. Temperature cycling conditions were performed in a Chromo 4 Detector (MJ Research, St. Bruno, Quebec, Canada) and included incubation for 5 min at 94°C, followed by 30 to 40 cycles at 94°C for 1 min, 57°C for 40 seconds, and 72°C for 50 seconds, and a final incubation at 70°C for 10 min. The primers used in these reactions were mouse-specific and have been described previously: Nanog [26], collagen type I [27], tenomodulin [28], LPL [29], Sox-9 [30] and Runx-2 [31]. These primers were obtained from Invitrogen (Grand Island, NY). Expression of each gene was normalized with respect to the expression level of glyceralehyde-3-phosphate dehydrogenase (GAPDH) [27]. Normalized values were then compared to the control group without mechanical stretching. Relative gene expression levels were calculated using the formula: 2^−ΔΔCT^ [32], where ΔΔCT = (CT_target gene_ − CT_GAPDH_)_mechanical stretching_ − (CT_target gene_ − T_GAPDH_)_control_ and CT represents the threshold cycle of each cDNA sample. Each reaction had 3 replicates and all 3 CT values were used to determine the mean ± standard deviation (SD) for each data set.

### Mouse treadmill running

For the treadmill running experiment, 18 aging mice (9 months old) were used. Mice were divided into two groups, a treadmill running group and a cage control group, with nine mice in each group. Mice in the treadmill running group were first trained for treadmill running by allowing to run 15 min/day for 5 days in the first week. They were then subjected to moderate treadmill running (MTR) at 50 min/day for 5 days in the following 3 weeks. In the cage control group, mice were allowed unrestricted movement in the cages. At the end of treadmill running, patellar tendons from all 18 mice in both control and MTR groups were dissected out and used for cell culture, histological and immunohistochemical analyses (see below).

### Culture of mouse TSCs after treadmill running

TSCs, isolated from the patellar tendons of 6 mice in the control and MTR groups each, were grown for 10–14 days in DMEM + 20% FBS, and monitored for colony formation and cell morphology through a microscope. Furthermore, semi-quantitation of the TSCs in culture was determined by manual identification of the cobblestone-shaped TSCs in the control and MTR groups through a Nikon eclipse microscope at 20x magnification. TSCs were counted from seven images obtained from various areas on the culture plates and further analyzed using the SPOT imaging software (Diagnostic Instruments, Inc., Sterling Heights, MI). The percentage of TSCs in a group was calculated by dividing the number of cobblestone-shaped TSCs by the total number of cells in the microscopic field. The average value of all seven images from each plate was used to represent the percentage of TSCs in that group.

In addition, population doubling time (PDT) of TSCs isolated from the control and MTR groups was calculated from the formula log_2_[Nc/N_0_] as described previously [22].

### Stem cell marker expression in mouse patellar tendons after treadmill running

Immunohistochemical analysis to identify the presence of the stem cell marker, NS, in TSCs from the patellar tendon sections and the subsequent semi-quantitation of the positively stained cells were performed as described above (See the section *Detecting non-tendinous tissues in tendons from young and aging mice*). Nuclei were counter-stained with Hoechst 33342 (1 μg/ml; Sigma, St. Louis, MO) at room temperature for 5 min.

### Histochemical analysis of mouse patellar tendons after treadmill running

Immediately after treadmill running, both patellar tendons from 3 mice in the control and MTR groups each were harvested and placed in frozen section medium (Neg 50; Richard-Allan Scientific; Kalamazoo, MI) in pre-labeled base molds. Then, they were flash frozen in 2-methylbutane chilled in liquid nitrogen and used immediately or stored at -80°C until further analysis. For histochemical analysis, the tissue blocks were sectioned (10 μm), dried overnight at room temperature and stained with Oil Red O, Safranin O or Alizarin Red S as described previously [22]. Finally, positive staining was visualized through a fluorescence microscope (Nikon eclipse microscope, TE2000-U). The positively stained regions in the tendon sections were quantified as described above (See section *Detecting non-tendinous tissues in tendons from young and aging mice* above).

### Statistical analysis

Statistical analysis of all data was performed using a two tailed *t*-test or one-way ANOVA followed by Fisher’s PLSD test for multiple comparisons. Differences between control and mechanical stretching or MTR groups were considered to be significant when *P*-values were less than 0.05.

## Results

### TSC proliferation depends on mouse age

**Fig 1:**
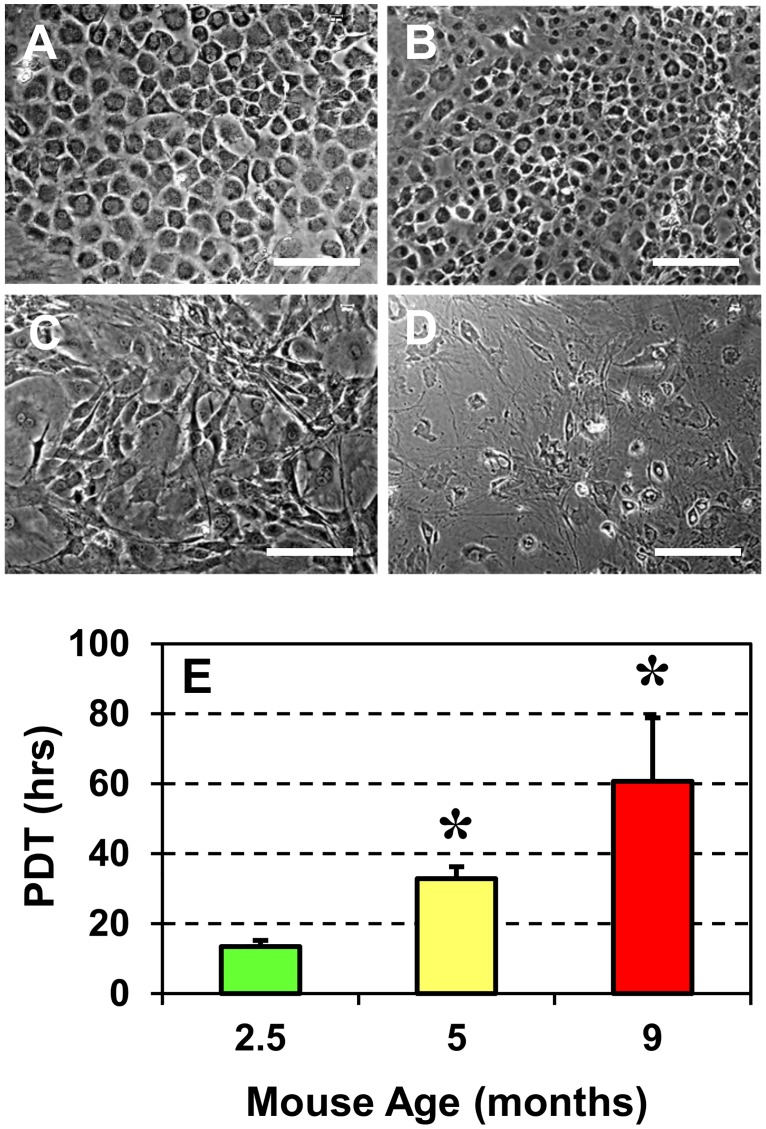
Morphology and proliferation of TSCs derived from young and aging mice. **A**. 2.5 months, **B.** 5 months, **C.** 9 months, and **D.** 24 months. With increasing mouse age, TSC morphology changed from cobblestone shape (**A**, **B**) to elongated/irregular shape (**C**, **D**). **E.** Proliferation of young and aging TSCs in culture measured by the population doubling time (PDT). The data shows that older the mice, greater the PDT or slower the TSC proliferation. Data represent the mean ± SD of three values. Statistical significance was calculated using the Student’s *t*-test (*P < 0.05) with data from the 2.5-months old mice used as reference. Bar—100 μm.

The morphology of TSCs isolated from mice aged 2.5, 5, 9 and 24 months, and cultured in growth medium for 2 weeks was different between the young and aging groups (Fig 1A–1D). In this study, we grouped 2.5 and 5 months old animals as young mice and the 9 and 24 months olds as aging mice. TSCs from young mice (2.5 and 5 months old) grew densely and were homogenously cobblestone-shaped (Fig 1A and 1B), which is typical for "normal TSCs" in culture [22]. However, as age of the mice increased, the cell shape was heterogeneous and the numbers of cobblestone-shaped cells drastically declined (Fig 1C and 1D) with the oldest mice (24 months) having only a few TSCs exhibiting the cobblestone shape (Fig 1D). Similarly, microscopic observations showed that the growth of TSCs was higher in 2.5 months old mice but decreased gradually with increasing age, and the 24 months old mice had the lowest total number of cells (Fig 1D). The measurement of population doubling time (PDT) also confirmed visual observations and revealed a significant reduction in the proliferative potential of TSCs (Fig 1E). This increase in PDT was age-dependent: PDT was 14 hrs in TSCs from 2.5 months old mice, 33 hrs in 5 months old mice (1.4-fold more than 2.5 months old mice) and 61 hrs in 9 months old mice (3.5-fold higher than 2.5 months old). PDT of TSCs from 24 months old mice is not shown because only a few TSCs were isolated from this group and they were insufficient to establish an *in vitro* culture.

### Aging alters stem cell marker expression in mouse TSCs

**Fig 2:**
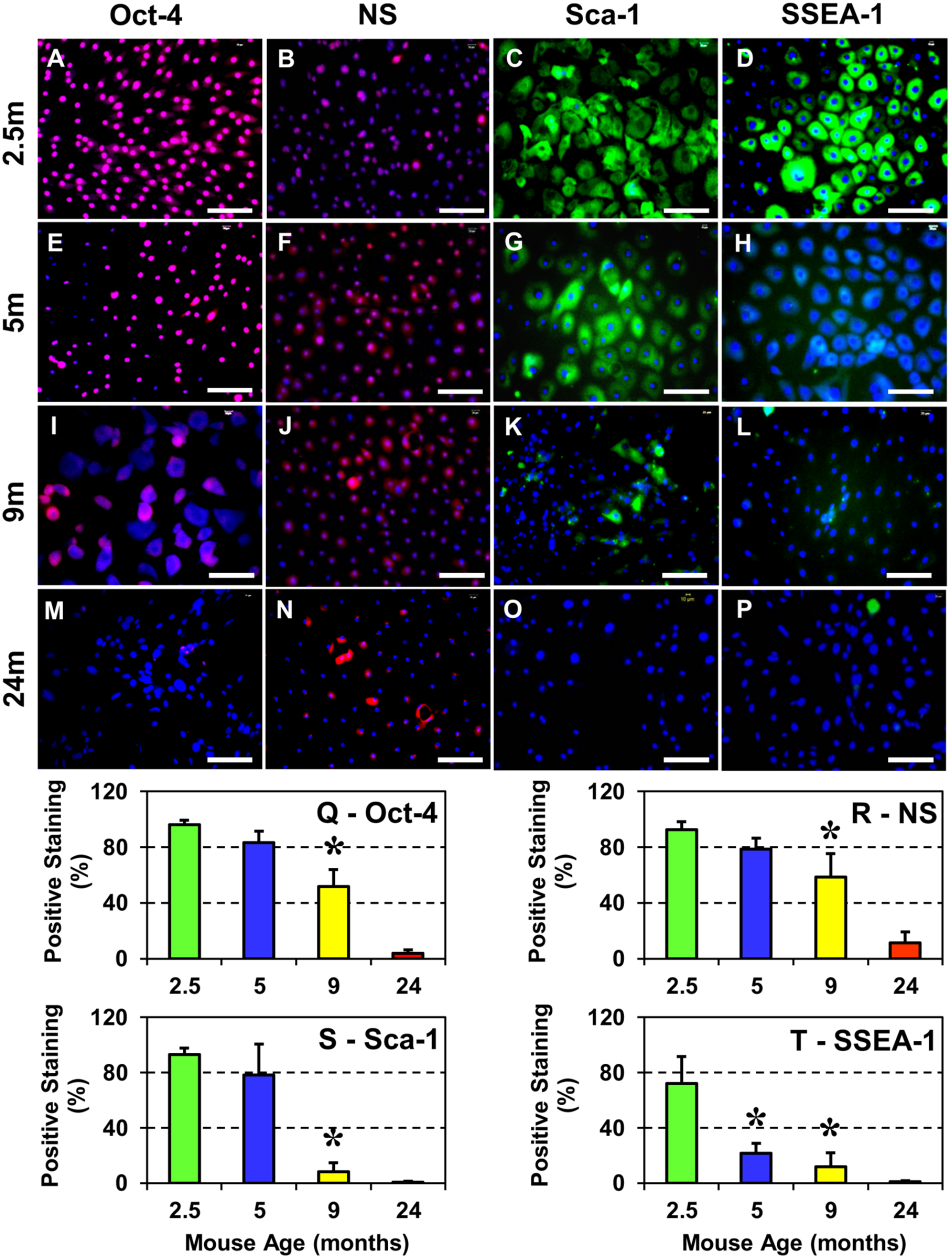
Stem cell marker expression in TSCs from differently aged mice (2.5m–2.5 months; 5m–5 months; 9m–9 months and 24m–24 months). **A–P:** Immunocytochemical staining for the indicated stem cell markers was performed using stem cell marker specific antibodies. **Q–T:** Semi-quantitation of stem cell marker expression after immunocytochemical staining. Increase in the mouse age decreased the number of TSCs expressing Oct-4, NS, Sca-1, and SSEA-1. In 24 months old mice, except for a few NS-expressing TSCs, expression of the remaining three stem cell markers (Oct-4, Sca-1 and SSEA-1) was very low. Semi-quantitation data are expressed as mean ± SD. Student’s *t*-test was used to determine statistical significance (*P < 0.05) in comparison with data from the 2.5-months old mice. Bar—100 μm.

To determine the effect of aging on the stemness of TSCs, the levels of four stem cell markers, Oct-4, NS, Sca-1 and SSEA-1, were analyzed by immunocytochemical staining. In previous studies these four markers were found to be expressed at high levels by TSCs in early passages [22,25] and therefore can be used to characterize the stemness of TSCs in culture. Examination of positively-stained TSCs revealed the robust presence of stem cell markers Oct-4, NS, Sca-1 and SSEA-1 in TSCs that were derived from 2.5 months old mice (Fig 2A, 2B, 2C and 2D). However, the extent of Oct-4, NS, and Sca-1 expression declined gradually from 2.5 months to 9 months old TSCs while the decrease in SSEA-1 levels was drastic and dropped significantly from 2.5 months to 5 months (Fig 2D and 2H). In 24 months old mice, only a few TSCs expressing NS could be identified (Fig 2N); the expression of other stem cell markers (Oct-4, Sca-1, and SSEA-1) were minimal (Fig 2M, 2O and 2P). Further analysis of the positively-stained cells by semi-quantification corroborated the microscopic observations and confirmed the effect of aging on stem cell marker expression in TSCs; Oct-4, NS, and Sca-1 staining decreased by ~15% in 5 months (Fig 2Q, 2R and 2S), and by ~47% (Oct-4), or ~37% (NS) in 9 months old mice (Fig 2Q and 2R). However, there was a sharp decline (~90%) in the levels of Sca-1 from 5 to 9 months and barely any staining was detected at 24 months (Fig 2S). SSEA-1 staining declined by ~70% in 5 months old mice, ~84% in 9 months and ~ 99% in 24 months old mice when compared to younger mice (2.5 months old) (Fig 2T).

Together, the results of population doubling time and immunostaining of stem cell marker expression in TSCs from differently aged mice show that 24 months old aged mice are not ideal for this study since they have low numbers of TSCs as well as express negligible amounts of Oct-4, Sca-1 and SSEA-1, which will likely limit the meaningful interpretation of experimental data after *in vitro* and *in vivo* “exercises”. Therefore, to represent the aging mice group we used the 9 months old mice in the following experiments.

### Presence of non-tendinous tissues in tendons of aging mice

**Fig 3:**
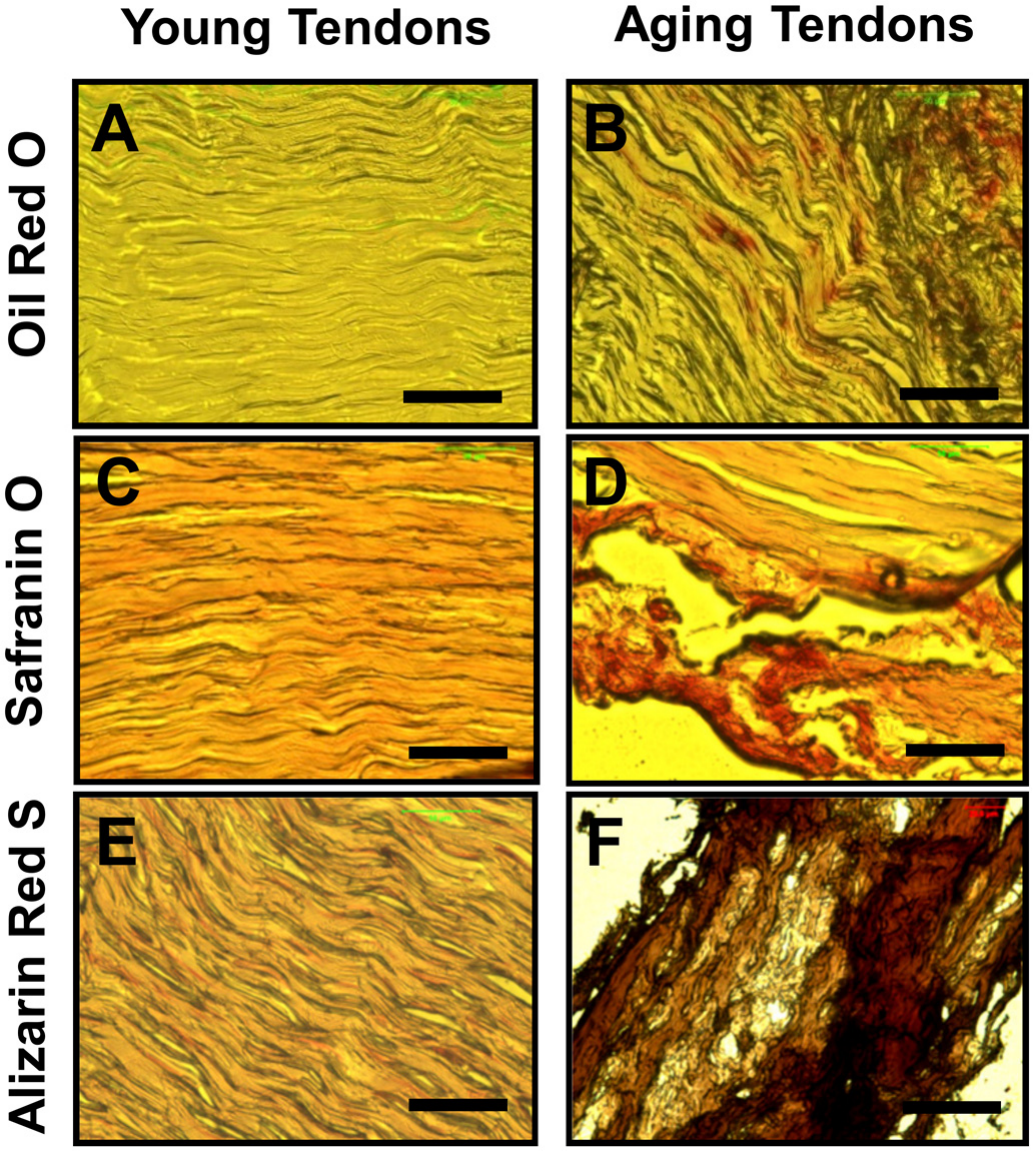
Histochemical analysis of patellar tendon sections from young (2.5 months) and aging (9 months) mice. Tendon sections were stained with Oil Red O (**A, B**), Safranin O (**C, D**) and Alizarin Red S (**E, F**) to detect the presence of lipids, proteoglycans, and calcium deposits, respectively. As shown, only minimal amounts of these non-tendinous tissues were detected in young tendons; in contrast, extensive amounts of these tissues were found in aging tendons. Bar—50 μm.

To investigate whether aging affects mouse tendons, we used two approaches. First, we used histochemical staining to determine whether tendons contained non-tendinous tissues. The results showed that the patellar tendons from young mice stained minimally for fatty-tissues (Fig 3A), proteoglycans (Fig 3C) and calcium (Fig 3E), compared to the tendons from aging mice, which had higher accumulation of the three non-tendinous tissues (Fig 3B, 3D and 3F). Evidently, the presence of non-tendinous tissues in these tendon sections from aging mice compromised tendon structure (Fig 3B, 3D and 3F).

**Fig 4:**
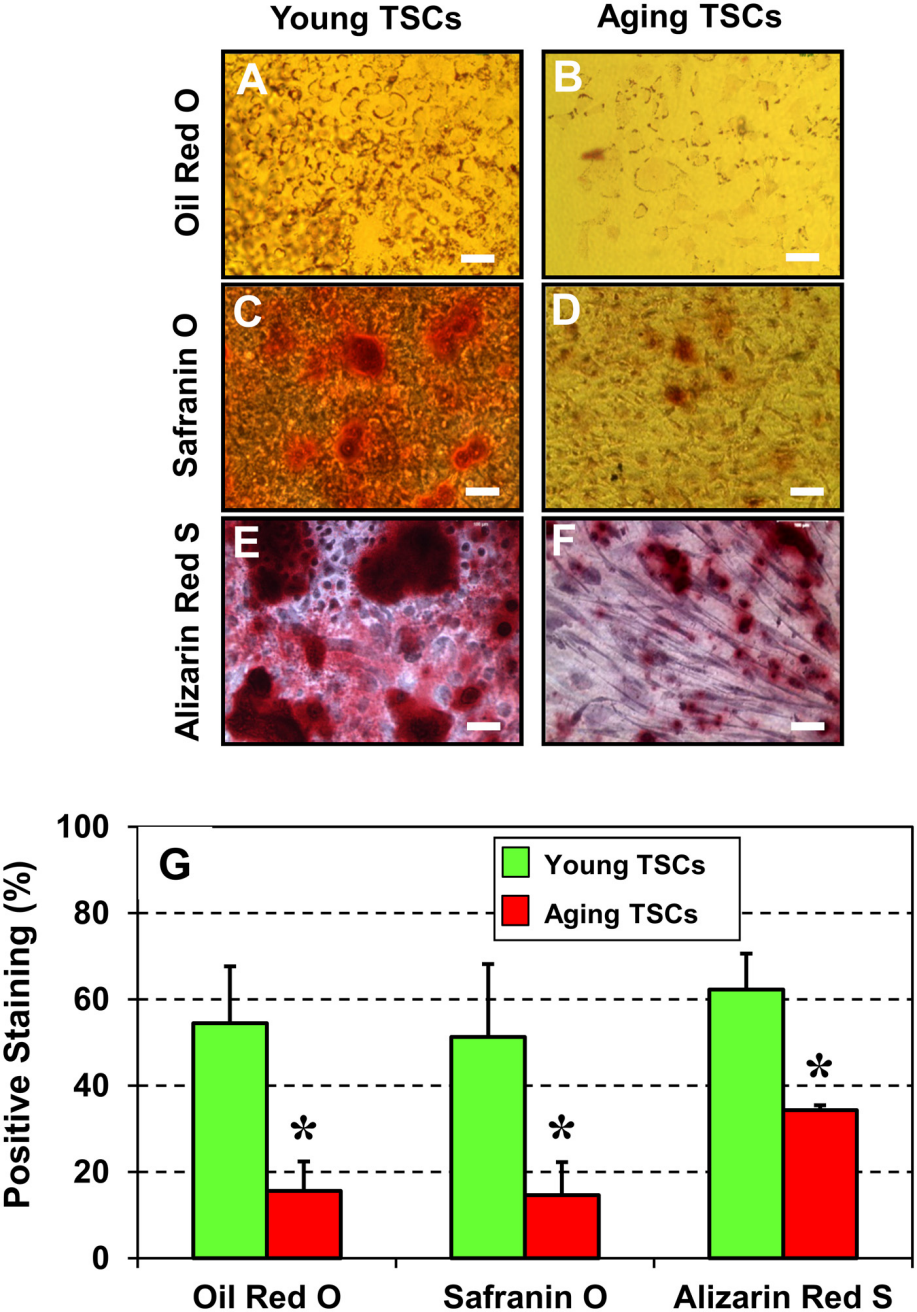
Cytochemical analysis of the non-tenocyte differentiation of TSCs from young (2.5 months) and aging (9 months) mice (G-L). TSCs were analyzed for their ability to undergo multi-differentiation by incubating in specific differentiation induction media and staining with Oil Red O (**G**, **H**), Safranin O (**I**, **J**) and Alizarin Red S (**K**, **L**). Evidently, young TSCs in culture differentiated more extensively into adipocytes (**G**), chondrocytes (**I**), osteocytes (**K**) than their counterparts—aging TSCs (**H**, **J**, **L**). Semi-quantitation (**M**) of the positively stained regions was performed by analyzing 12 different images of each tendon section. Data are mean ± SD, and *P < 0.05, compared to young TSCs.

Our second approach was *in vitro*, where we induced TSCs, isolated from young and aging mice, to differentiate into non-tenocytes by culturing the cells in non-tenogenic induction media. Cytochemical staining showed that young TSCs stained heavily for Oil Red O (Fig 4A), Safranin O (Fig 4C) and Alizarin Red S (Fig 4E) compared to TSCs from aging mice (Fig 4B, 4D, 4F). Semi-quantification (Fig 4G) also confirmed such an increase and further revealed that the extent of Oil Red O and Safranin O staining in young TSCs were higher by ~3-fold each and that Alizarin Red S staining was higher by ~1.5 fold. These results indicate that when compared to young TSCs, aging TSCs have lower potential to differentiate into non-tenocytes *in vitro* suggesting a decline in the quality of TSCs in aging mice.

### *In vitro* mechanical loading increases the number of NS positive TSCs and modulates tenocyte and non-tenocyte related gene expression

**Fig 5:**
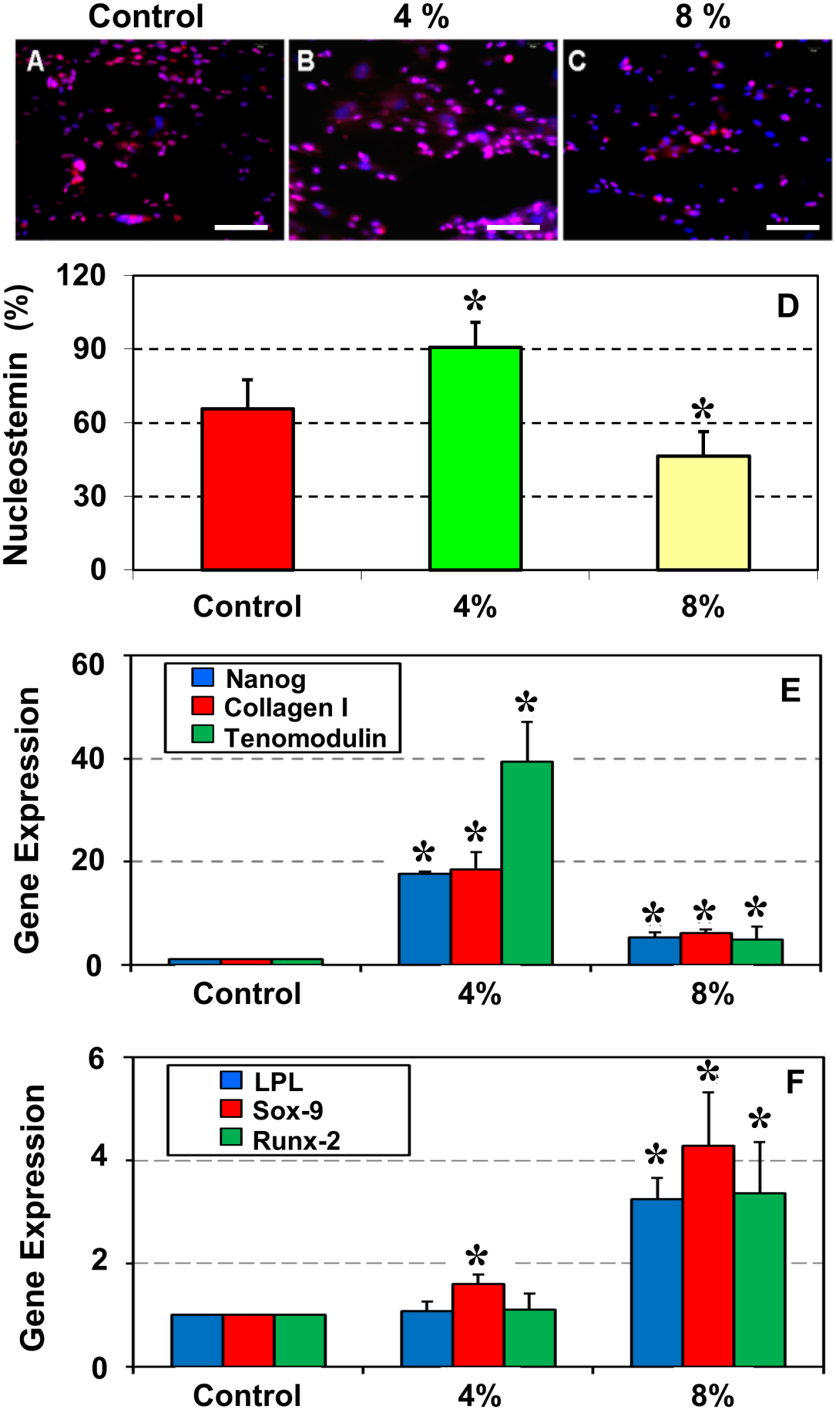
Immunostaining of nucleostemin (NS) (A–C), semi-quantitation (D) and qRT-PCR analysis of gene expression (E, F) in aging TSCs subjected to mechanical loading *in vitro*. Patellar TSCs isolated from aging mice (9 months old) were subjected to no stretching (Control), 4% stretching or 8% stretching followed by the analyses. Immunostaining (**A–C**) and semi-quantitation (**D**) showed that compared to the control without stretching, 4% stretching increased the number of NS-expressing TSCs, whereas 8% stretching further decreased it. qRT-PCR analysis of the expression of three stem cell and tenocyte-related genes, Nanog, collagen I and tenomodulin (**E**) and three non-tenocyte related genes, LPL, Sox-9 and Runx-2 (**F**) showed that 4% stretching effectively up-regulated Nanog, collagen I and tenomodulin, but 8% was less effective (**E**). Moreover, while 4% stretching did not alter LPL and Runx-2 expression, and slightly up-regulated Sox-9 level, 8% stretching up-regulated the expression of all three genes (**F**). Data are mean ± SD, and *P < 0.05, compared to the respective controls. Bar—100 μm.

To evaluate the effect of mechanical loading on aging, TSCs isolated from aging mice (9 months) were subjected to two levels of stretching that corresponded to low (4%) and high (8%) magnitudes. Such cyclic mechanical stretching *in vitro* was specifically designed to mimic *in vivo* mechanical loading conditions [24]. After the mechanical loading, immunocytochemical staining for NS was performed. Compared to the control (Fig 5A), the 4% stretched cells showed increased staining for NS (Fig 5B). However, after 8% stretching (Fig 5C) staining for NS was similar to the control (Fig 5A). Semi-quantitation of the NS positive cells showed that when compared to the control, 4% stretching led to a 38% increase in NS-positive TSCs, whereas 8% stretching resulted in a 29% decline in NS-positive TSCs (Fig 5D).

Furthermore, the effect of mechanical loading on aging TSCs was determined by gene expression analysis using qRT-PCR. We found that expression levels of the tenocyte-related genes, Nanog, collagen I, and tenomodulin, remained low in the control TSCs without mechanical loading. When TSCs were subjected to 4% stretching there was a significant increase in the net expression levels of all three genes over the control: Nanog increased by 17-fold, collagen I by 18-fold, and tenomodulin by 37-fold. However, 8% stretching had a smaller effect on gene expression: it increased the expression of all three genes by 6-fold compared to the control (Fig 5E).

Moreover, expression of the non-tenocyte related genes, LPL (adipocyte), Sox-9 (chondrocyte), and Runx-2 (osteocyte), was determined in aging mouse TSCs subjected to mechanical loading. These results differed from the tenocyte-related genes: 4% stretching did not alter the expression of LPL or Runx-2, and only slightly increased Sox-9 levels; however, 8% stretching up-regulated all three non-tenocyte genes; LPL and Runx-2 increased by ~2-fold each, and Sox-9 by ~4-fold over the control (Fig 5F).

### Moderate treadmill running (MTR) enhances TSC numbers and function in aging tendons

**Fig 6:**
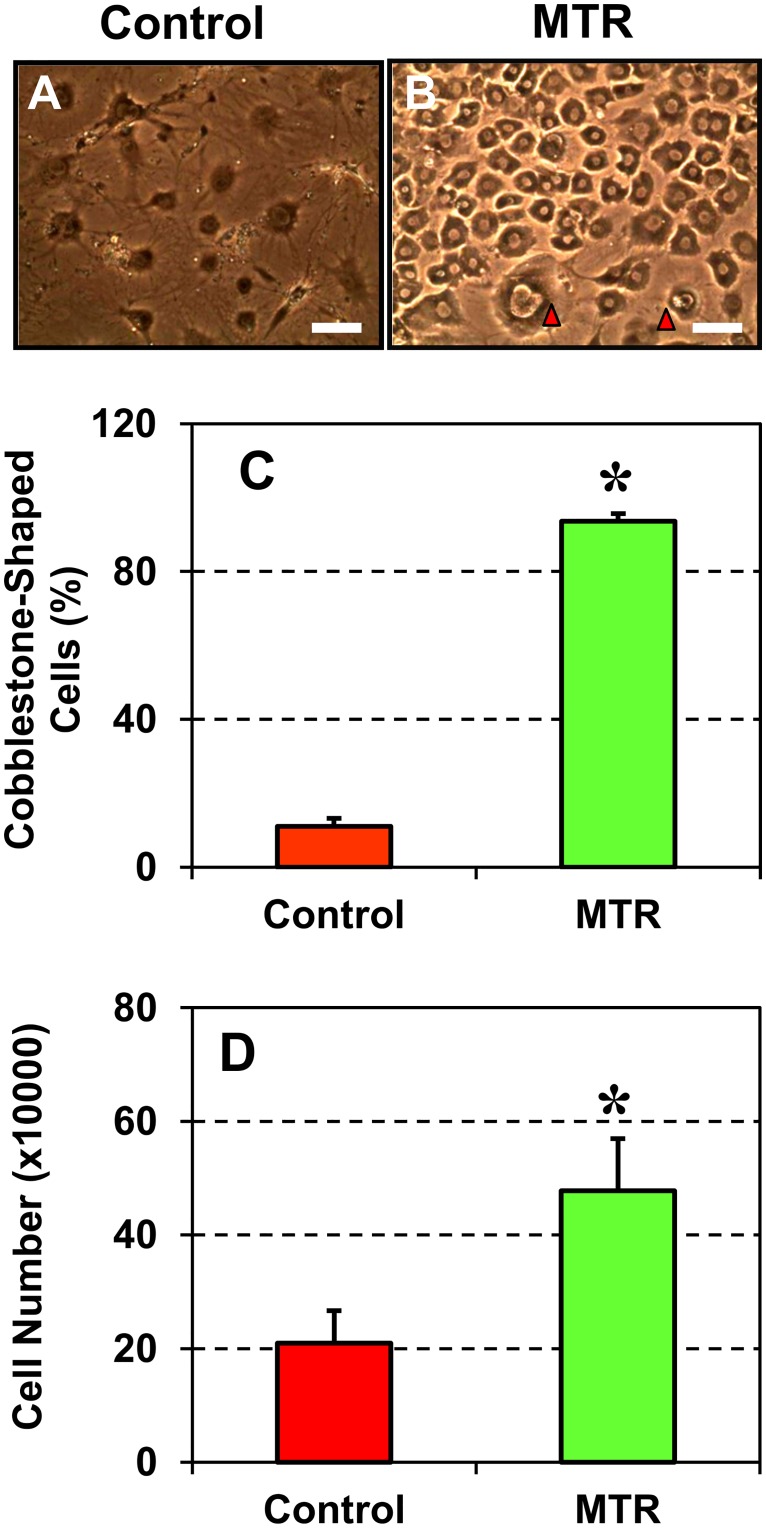
Morphology and proliferation of TSCs from aging mice after moderate treadmill running (MTR). **A.** Control mice without MTR. **B**. Mice after MTR, **C.** Semi-quantitation of the number of cobblestone-shaped TSCs. **D.** TSC proliferation, measured as PDT, from mice in the control and MTR groups. Aging TSCs in the control group were large and round, a typical phenotype of senescent cells, but after MTR most cells exhibited a cobblestone shape (arrows point to the two remaining large TSCs as in the control group), which also proliferated at a higher rate. Data are mean ± SD, and *P < 0.05, in comparison with data from the control group. Bar—100 μm.

The effects of mechanical loading on TSCs *in vivo* was investigated by subjecting aging mice (9 months old) to run on a moderate treadmill running (MTR) regimen, which was previously shown to induce beneficial effects in tendons [21,25]. TSCs isolated from the control group without treadmill running were sparse in culture and were round in shape (Fig 6A). In contrast, TSCs subjected to MTR exhibited a cobblestone-shaped structure that is typical for active TSCs (Fig 6B). Semi-quantitation further supported these observations and showed that MTR increased the number of cobblestone-shaped cells by 88% (Fig 6C). Further, TSCs from mice in the MTR group proliferated significantly quicker in cultures when compared to those from the control group (Fig 6D). After five days in culture, the number of TSCs in the MTR group was 1.3-fold more than the TSCs numbers in the control group.

### Moderate treadmill running (MTR) increases NS-expressing TSCs and reduces non-tendinous tissue formation in aging mouse tendons

**Fig 7:**
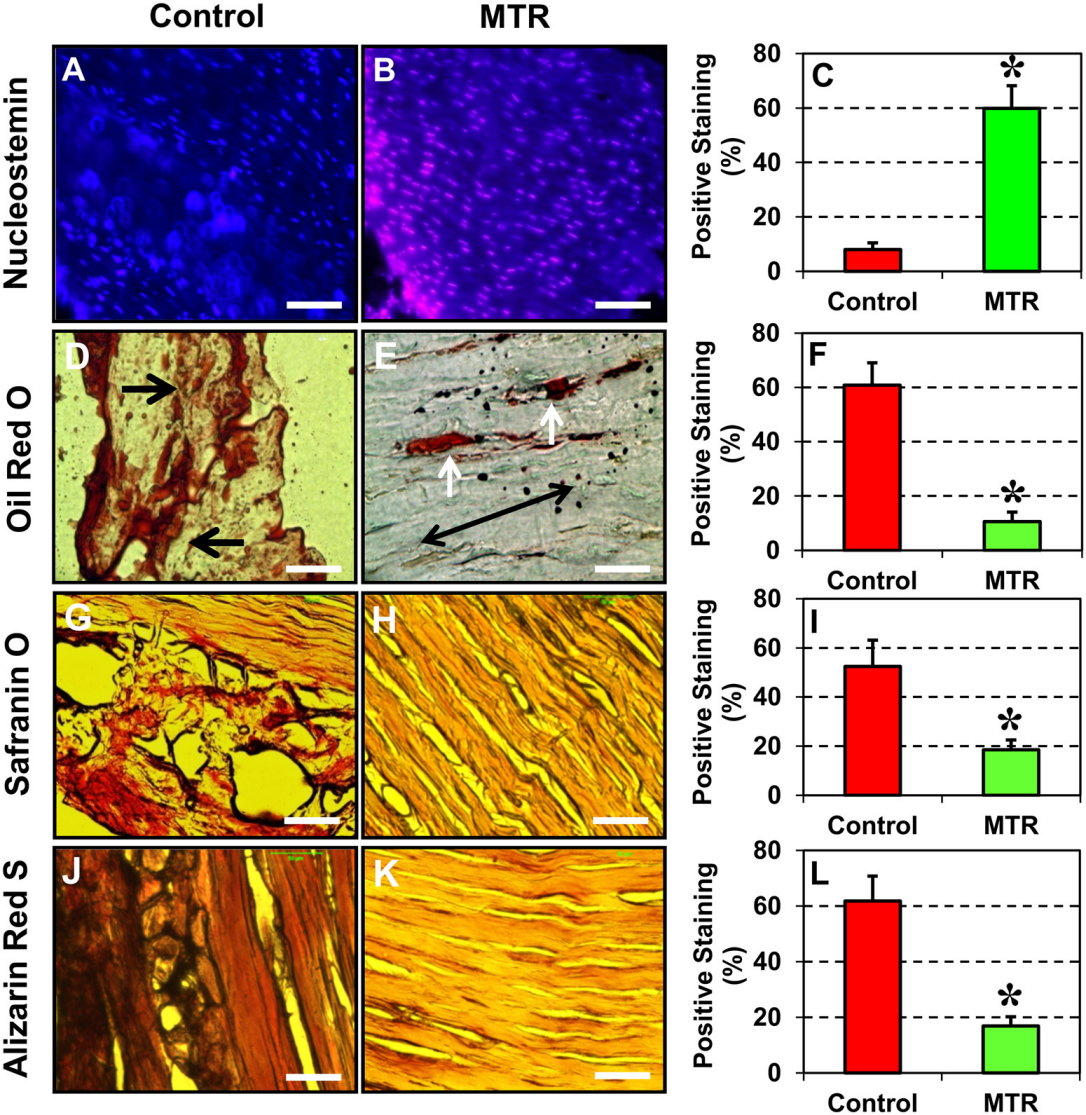
Effect of moderate treadmill running (MTR) on NS expression (A–C) and multi-differentiation potential (D–L) of TSCs in aging mice (9 months). Immunostaining indicated that MTR significantly increased the number of NS-expressing TSCs compared to the control without treadmill running (**A, B, C**). Histochemical staining was also performed to analyze the ability of aging tendon cells without treadmill running (**D, G, J**) and after MTR (**E, H, K**) to undergo adipogenic, chondrogenic and osteogenic differentiation by staining with Oil Red O (**D, E, F**), Safranin O (**G, H, I**) and Alizarin Red S (**J, K, L**), respectively. Extensive lipids (**D**, arrows point to accumulated lipids), proteoglycans (**G**), and calcium deposits (**J**) were present in the control tendons. After MTR, less non-tendinous tissues were found in the tendons (**E**, two white arrows point to a few residual lipids, and a double arrow indicates the long axis of the tendon). The nuclei stained with Hoechst 33342 appear blue. Semi-quantitation confirmed the histochemical analysis. Data are mean ± SD, and *P < 0.05, compared to the respective controls. Bar—50 μm.

In the control aging mice, very few NS-expressing TSCs were present in the patellar tendons (Fig 7A); however, after MTR, numerous NS-expressing TSCs were identified (Fig 7B). Semi-quantitation also showed a 6.5-fold increase in the number of NS-expressing TSCs in the MTR group when compared to the control (Fig 7C).

Histochemical analysis of the patellar tendons from aging mice showed large amounts of lipid deposits (Fig 7D), proteoglycan accumulation (Fig 7G) and calcium deposits (Fig 7J) within tendon tissues. However, after mice of the same age ran on the treadmill, a marked reduction in all three non-tendinous tissues were observed: amounts of lipid deposits decreased (Fig 7E), and only small amounts of proteoglycans (Fig 7H) and calcium deposits (Fig 7K) were present in the patellar tendons. It should be pointed out that the sections of aging tendons were easily distorted during sectioning because of the extensive degenerative changes (e.g. extensive lipid accumulation) (Fig 7D, 7G and 7J). Semi-quantitation of the positive staining also confirmed these observations and further revealed that lipid deposition decreased by 83%, proteoglycan by 65% and calcium deposition by 73% in the patellar tendon sections from mice in the MTR group (Fig 7F, 7I and 7L).

## Discussion

Tissue-specific stem cells are essential for the maintenance, repair, and regeneration of tissues in vertebrates. In tendons, TSCs, which are the tendon-specific stem cells maintain tendon homeostasis and repair once they are injured [22]. It is known that aging weakens connective tissues such as tendons in the human body and that appropriate mechanical loading via exercise can strengthen them. Nevertheless, how aging affects TSCs and how mechanical loading impacts aging tendons at the TSC level are not well understood. In this study, we have shown that aging causes detrimental effects on mouse TSCs by decreasing their proliferation and reducing their stemness *in vitro*; on the other hand, “exercise” through moderate mechanical stretching (4%) increased the proliferation, stemness, and tenocyte-related gene expression in TSCs. Furthermore, moderate treadmill running (MTR) also decreased tendon degeneration manifested by lipid deposition, proteoglycan accumulation, and calcification in the affected tendons, and increased the number of TSCs and their stemness *in vivo*. These findings support our hypothesis that aging impairs the function of TSCs, while moderate exercise improves the "health" of aging tendons likely by enhancing TSC proliferation and differentiation into active tenocytes.

Additionally, we also found that excessive mechanical stretching (8%) of aging TSCs *in vitro* increased non-tenocyte related gene expression similar to our previous findings on young mouse (2.5 months) TSCs [21]. This indicates that while moderate exercise is beneficial for both young and aging TSCs, excessive mechanical loading is detrimental to TSCs of all ages.

Our findings in mouse TSCs are in line with previous findings on TSCs isolated from human hamstrings [5], human Achilles tendons [6], and rat TSCs [7]. These studies reported that fewer TSCs were present in aging human and rat tendons compared to younger tendons, and that these TSCs from aging tendons had reduced potential for self-renewal and formation of colonies in culture.

In addition, by performing histochemical analysis on tendon sections *in vivo* we identified little non-tendinous tissues in young mouse patellar tendons but the presence of abundant lipid, proteoglycan and calcium deposits in aging mouse tendons (Fig 3), which are characteristics of degenerative tendinopathy [13]. In contrast, our *in vitro* studies showed that when induced, young TSCs could differentiate more extensively into adipocytes, chondrocytes and osteocytes compared to aging TSCs (Fig 4) indicating that TSCs from young mice are of higher quality than TSCs from aging mice. This suggests that young tendons likely contain more potent stem cells than aging tendons, which is evident by the higher expression of the stem cell markers, NS, Oct-4, Sca-1, and SSEA-1 in young TSCs when compared to aging TSCs (Fig 2). However, despite the ability of young TSCs for higher multi-differentiation potential, degenerative changes such as lipid deposits, proteoglycan accumulation and calcium deposits were not observed in young tendon sections. This finding indicates that TSCs in young tendons in general do not exhibit aberrant differentiation likely due to the conducive tenogenic environment, including low oxygen tension inside the tendon, which helps maintain the stemness of TSCs [21]. Other factors that may create a tenogenic environment include intact tendon extracellular matrix, as well as a lack of excessive mechanical loading. On the other hand, degenerative changes were extensive in aging tendons (Fig 3), which could be at least in part due to the poor quality of TSCs that may undergo aberrant non-tenocyte differentiation due to aging (Fig 4). These results indicate that aging is a major risk factor for the development of degenerative tendinopathy in old patients, which typically manifests by the formation of non-tendinous tissues in the tendon [13].

In addition to aging, our previous studies have shown that excessive mechanical loading of mice also induces high level expression of non-tenocyte related genes, LPL, Runx-2, Sox-9 and Osterix [21,25], which can lead to aberrant differentiation of TSCs into non-tendinous tissues. Further, hTSCs exposed to high levels of PGE_2_ also expressed high levels of non-tenocyte markers PPARγ, collagen II and osteocalcin, when implanted into rats [33] and lead to non-tendinous tissue formation. Taken together, these findings suggest that factors such as aging, excessive exercise and high PGE_2_ levels can induce aberrant differentiation of TSCs in otherwise healthy tendons.

Interestingly, reports from previous studies on the age-related differentiation potential of TSCs are not consistent. For example, Zhou et al. reported that both young and aging rat TSCs can differentiate into chondrocytes and osteoblasts; however, more aging TSCs differentiate into adipocytes than young TSCs [7]. These aging TSCs also expressed higher levels of the adipogenic marker genes, PPARγ2, C/EBPa, and leptin. In contrast, a study on human TSCs found that while both young and aging TSCs differentiated equally into all three non-tenocyte lineages, the mRNA levels of PPARγ (adipogenesis) and Runx-2 (osteogenesis) were up-regulated in younger TSCs (20–22 years old adults), while Sox-9 (chondrogenesis) was highly expressed in the older age group (49–50 years old adults) [5]. Yet another study did not find any obvious difference between young and aging human TSCs’ ability to differentiate in the three non-tenocyte lineages, adipocytes, chondrocytes and osteocytes [6]. The differences in all these studies including ours could be attributed to various factors including potential differences in culture conditions, induction media used, animal age and species-specific differences, among others.

Although a number of studies have reported the presence of non-tendinous tissues in aging tendons, the source of these non-tenocytes has not been well studied. In a previous *in vitro* study, we showed that non-tenocytes were formed only when TSCs but not tenocytes from young mice (2.5 months) were subjected to high mechanical stretching (8%) [21]. A recent *in vivo* study also confirmed these findings; in this study, we first eliminated native tendon cells by irradiating mice tendons followed by injection of GFP-tagged TSCs into the region. Evaluation of the tendon sections five days later showed that the region was re-populated with GFP positive TSCs. Furthermore, when these mice were subjected to intensive treadmill running, GFP-expressing non-tenocytes were observed in the irradiated and injected region [34]. These findings indicate that non-tenocyte differentiation of TSCs is one mechanism likely responsible for the development of aging-associated tendinopathy. However, since tendinopathy is a spectrum of tendon disorders [35], other factors, such as infiltration of external cells, alteration in the tendon matrix composition and organization, and damages to the tendon matrix, could also play a role in the development of tendinopathy in aging animals/patients.

We found that the ability of aging TSCs to proliferate decreased in an age-dependent manner when cultured *in vitro*. Indeed, when compared to young mice (2.5 and 5 months) much fewer TSCs could be isolated from 24 months old mice indicating a decline in the numbers of TSCs in aging mice. Previous studies on human TSCs also showed that aging decreased the number of isolated TSCs and their proliferation potential [5]. Although the molecular mechanisms underlying the decreased proliferation in aging TSCs is not clear, it has been shown that tenocytes from the Achilles tendons of aging rats had reduced levels of collagen I, collagen I/III ratio, aggrecan and elastin [36]. Similarly, the down-regulation of cellular senescence-inhibited gene and up-regulation of p27 [37], a cellular senescence-related marker, was associated with the decreased proliferation of tenocytes in aging tendons. Concomitant with decreased proliferation, the expression levels of tendon-lineage marker genes, scleraxis and tenomodulin, were reduced while adipocytic differentiation was increased along with increased accumulation of the adipocytic marker gene, PPARγ, in aging rat TSCs [7]. Finally, in aging rat TSCs a reduction in the expression of the transcription factor, Cited2, was also reported [7]. Cited2 has been shown to play a role in the maintenance of adult hematopoietic stem cells [38], proliferation of mouse embryonic fibroblasts [39], and down-regulation of shear-induced MMP-1 and MMP-13 production in human chondrocyte cell lines [40]. Since TSCs play a critical role in tendon maintenance and repair, these data may explain why aging individuals tend to develop degenerative tendinopathy and why treatment of tendon injuries in aging individuals is challenging, simply because of altered TSC function at the cellular and molecular levels.

Previously, it was reported that more than 96% of mouse TSCs expressed the stem cell marker, Sca-1 [2]. We made similar observations in young 2.5 months old mice TSCs in this study. In addition to Sca-1, the results presented here and our previous studies also showed the expression of other stem cell markers, Oct-4, Nanog, and NS in young mouse TSCs *in vitro* [21,22]. In this study, we found that the expression of all these stem cell markers (Sca-1, NS, Oct-4, and Nanog) decreased in an age-dependent manner although the function of these markers in TSC's biological activities is still unclear. The expression of NS however, was enhanced in aging TSCs subjected to mechanical stretching *in vitro* and in patellar tendons from aging mice that ran on a treadmill when compared to their respective control groups without moderate exercise. It should be noted that among the multiple markers available to identify TSCs, we used NS, which is however a general marker of stem/progenitor cells; therefore, the high level expression of NS in the MTR group represents an increase in the total stem/progenitor cells of which TSCs could be only a sub-population, albeit a large one. In addition, we found that moderate exercise in the form of treadmill running suppressed degenerative changes (lipid deposits, proteoglycan accumulation and calcification) (Fig 7) and increased the number of TSCs in aging mouse tendons (Fig 6). Moderate stretching (4%) also induced beneficial effects by increasing the expression of tenocyte related genes, Nanog, collagen I and tenomodulin (Fig 5E). These results are consistent with our previous findings that moderate exercise increases tenocyte related gene expression while intense exercise increases both tenocyte and non-tenocyte related gene expression in young mouse tendons *in vivo* and TSCs *in vitro* [21,22]. Taken together, these findings suggest that moderate exercise, such as MTR, exerts beneficial effects on aging tendons by increasing the number of TSCs and promoting the appropriate differentiation of TSCs into tenocytes.

In this study, we used 9 months old mice to run on our MTR protocol. It is presumed that choosing aging mice (9 months) instead of aged mice (> 24 months) allowed the successful completion of the MTR regimen. In addition, the number of TSCs in 24 months old mice was very low for the MTR regimen to significantly impact TSC proliferation and non-tendinous tissue formation (lipid deposits, proteoglycan accumulation and calcium deposits) in the patellar tendons of mice subjected to MTR. A number of aging-related studies have used very old mice (> 24 months) to understand aging related changes in tendons [7,41]. Ours is one of the few studies to use aging mice (9 months) to study the moderate exercise induced beneficial changes in aging tendons. Recently, the presence of calcium deposits was reported in the Achilles tendons of 8 months old mice [41] thus supporting the presence of degenerative changes in the tendons of 8–9 months old aging mice. Interestingly, tendon "degeneration" already occurs in 5 months old mice, in terms of declining TSC number and stem cell marker expression when compared to younger mice (2.5 months) (Figs 1 and 2). While it is expected that older mice (> 9 months) may have severe degenerative changes, the use of 9 months old mice in this study facilitated observation of the beneficial effects of moderate exercise in a more consistent manner.

A limitation of this study is that we determined only the effects of MTR but not the effects of intense treadmill running (ITR) on aging tendons and TSCs. Previously we showed that ITR induced aberrant changes in young mouse tendons/TSCs *in vivo* in terms of non-tenocyte gene expression [21]. Therefore, it is likely that ITR will not be as effective as MTR in reducing impaired TSC function and tendon degeneration. An indirect support for this conjecture comes from our *in vitro* stretching experiments of TSCs. An 8% stretching, which is considered as a large stretching load on TSCs, markedly increased the expression of non-tenocyte related genes in TSCs (Fig 5F) that may lead to non-tendon tissue formation. Future studies should address the effects of ITR on aging tendons. In addition, since this study is meant to be a proof-of-concept study, we determined the effects of MTR only at one time point and did not evaluate the functional outcomes of aging mouse tendons after MTR. These additional variables will be addressed in a future study. Furthermore, while our results demonstrate that MTR reduced the detrimental effects of aging on TSCs due to increased proliferation ability, enhanced expression levels of tenocyte genes, and reduced degenerative changes in aging tendons, the precise molecular mechanisms by which MTR enhances TSC function that is translated into the beneficial effects of MTR on aging tendons are not clear. Lastly, these findings on mouse tendons may not be directly translated to humans because of the vast differences in physiology/activities between the two mammals and also due to the fact that young adult mice do not spontaneously develop tendinopathy or tendon ruptures like humans due to lack of excessive mechanical loading such as those experienced by athletes. However, since aging mouse tendons develop degenerative changes similar to aging humans, similarities can be inferred from this study to suggest rehabilitation protocols for tendinopathy patients.

## Conclusions

In summary, this is the first study to determine the effects of moderate exercise on aging TSCs using *in vitro* and *in vivo* models. The findings of this study show that aging impaired the proliferative potential of TSCs, expression of stem cell markers and multi-differentiation potential of TSCs in an age-dependent manner. *In vivo*, aging also led to the formation of fatty tissues, proteoglycan accumulation and calcium deposition in tendons. On the other hand, MTR produced beneficial effects, namely, enhanced stemness of aging TSCs and decreased degeneration in aging tendons. The findings of this study point out that TSC-based mechanism could in part execute the beneficial effects of moderate exercise on aging tendons; however, the mechanisms by which mechanical loading such as moderate exercise affects tendons’ cellular fate and function remain to be investigated. Finally, based on the findings of this study, it is suggested that exercise such as moderate treadmill running regimens could be used to slow down or prevent tendon degeneration due to aging.
